# A Systematic Review of the Role of Proprietary and Patent Medicine Vendors in Healthcare Provision in Nigeria

**DOI:** 10.1371/journal.pone.0117165

**Published:** 2015-01-28

**Authors:** Naomi Beyeler, Jenny Liu, Maia Sieverding

**Affiliations:** Global Health Group, Global Health Sciences, University of California San Francisco, San Francisco, California, United States of America; London School of Hygiene and Tropical Medicine, UNITED KINGDOM

## Abstract

**Background:**

Interventions to reduce the burden of disease and mortality in sub-Saharan Africa increasingly recognize the important role that drug retailers play in delivering basic healthcare services. In Nigeria, owner-operated drug retail outlets, known as patent and proprietary medicine vendors (PPMVs), are a main source of medicines for acute conditions, but their practices are not well understood. Greater understanding of the role of PPMVs and the quality of care they provide is needed in order to inform ongoing national health initiatives that aim to incorporate PPMVs as a delivery mechanism.

**Objective and Methods:**

This paper reviews and synthesizes the existing published and grey literature on the characteristics, knowledge and practices of PPMVs in Nigeria. We searched published and grey literature using a number of electronic databases, supplemented with website searches of relevant international agencies. We included all studies providing outcome data on PPMVs in Nigeria, including non-experimental studies, and assessed the rigor of each study using the WHO-Johns Hopkins Rigor scale. We used narrative synthesis to evaluate the findings.

**Results:**

We identified 50 articles for inclusion. These studies provided data on a wide range of PPMV outcomes: training; health knowledge; health practices, including drug stocking and dispensing, client interaction, and referral; compliance with regulatory guidelines; and the effects of interventions targeting PPMVs. In general, PPMVs have low health knowledge and poor health treatment practices. However, the literature focuses largely on services for adult malaria, and little is known about other health areas or services for children.

**Conclusions:**

This review highlights several concerns with the quality of the private drug retail sector in Nigeria, as well as gaps in the existing evidence base. Future research should adopt a more holistic view of the services provided by PPMV shops, and evaluate intervention strategies that may improve the services provided in this sector.

## Introduction

The private sector delivers a substantial portion of healthcare services in many low- and middle-income countries [1]. Across Sub-Saharan Africa, private providers, ranging from informal providers and traditional healers to privately owned specialty hospitals, provide roughly half of all child health services [2]. In particular, drug shops comprise a sizable portion (nearly 40%) of the private healthcare sector in the region [3] and provide between 15% and 83% of all child health services [4]. To address the role of the retail drug sector, several countries in Sub-Saharan Africa have included private drug retailers in national health interventions [5–7].

In Nigeria, owner-operated drug retail outlets, or patent and proprietary medicine vendors (PPMVs), are a main source of medicine for acute conditions [8]. National surveys show that PPMVs are the first source of care for between 8% and 55% of illnesses occurring among children under five [9–11]. Community-and state-level studies of care-seeking behavior have similarly found that PPMVs are the first source of care for up to 55% of under-five child illnesses, and provide services for 35% to 55% of adults seeking malaria treatment [12,13]. PPMVs are a particularly important source of care in rural and lower income communities [14–16]. However, there is wide variation in the percentage of care-seeking that takes place at PPMVs across child health conditions, including diarrhea, fever and cough, [9,17] as well as across geography for the same condition, for example malaria [12,18–20].

PPMVs are defined as “a person without formal training in pharmacy who sells orthodox pharmaceutical products on a retail basis for profit” [21]. They were established as a category of retailer by the Ministry of Health to provide a source of medicine in communities with limited access to essential health commodities [22]. An estimated 200,000 operated in the country as of 2005, far outnumbering the 2,639 retail pharmacies that were registered in the same year [22], and more than all other cadres of health worker in the country [23]. The density of pharmacists was estimated at 10.5 per 100,000 population in 2008, as compared to 40 physicians and 161 nurses; pharmacies also tend to be geographically concentrated in urban areas [23].

PPMV licensure does not require formal training in medicine or pharmacy [24]. Rather, many PPMVs complete an apprenticeship with a more senior PPMV before opening their own shop, and by convention are expected to have completed primary school [21]. Regulations permit PPMVs to sell a limited number of pre-packaged, over-the-counter medicines and medical products, but prohibit them from selling prescription medications (including antibiotics) or conducting invasive medical procedures (e.g. injections) [25]. In the area of family planning, PPMVs are permitted to sell condoms and oral contraceptive pills, but are not allowed to prescribe or sell oral contraceptives to first-time contraceptive users or users experiencing complications [26]. In contrast, retail pharmacists must have a formal degree in pharmacy and are permitted to sell prescription medications [27]. Official licensing of PPMVs and retail pharmacies is overseen by the Pharmacists Council of Nigeria (PCN) [22].

Given their numbers, market share, and presence in rural communities, PPMVs represent an important opportunity for improving the delivery of primary healthcare commodities and services. There is growing interest among policymakers and program implementers in Nigeria to further engage PPMVs in primary health care delivery, as evidenced by a number of recent national health initiatives and regulatory changes that explicitly address PPMVs. For example, in 2005, the Essential Medicines list that outlines the drugs PPMVs are permitted to dispense was amended to allow PPMVs to sell artemisinin-based combination therapies (ACTs) [25] after the national treatment guidelines for uncomplicated malaria were revised to recommend ACTs rather than chloroquine as the first-line treatment [28].

The Government of Nigeria has similarly committed to adding pediatric zinc and co-packaged zinc and oral rehydration salts (ORS) to PPMVs’ list of approved medications as part of the national Essential Medicines Scale-Up Plan. The Plan also includes implementation of continuous education for PPMVs to improve care for common childhood illness [8]. PPMVs have been listed as potential community-level implementers for the newly adopted national Integrated Community Case Management (ICCM) guidelines [29], and have been included in a pilot home management of malaria project under the National Malaria Strategic Plan [8]. However, a greater understanding of the current characteristics, knowledge and practices of PPMVs is needed to identify effective mechanisms for operationalizing PPMVs’ integration into these national initiatives, as well as to identify additional programmatic and policy strategies for improving health services in the PPMV sector.

Across many countries in sub-Saharan Africa, privately owned drug shops, akin to Nigeria’s PPMVs, have increasingly been recognized as important providers of health commodities. Two recent reviews were completed on the characteristics and practices of specialized drug shops and related interventions in sub-Saharan Africa [30,31]. These reviews found highly variable quality and treatment practices at drug shops both within and across countries, and mixed evidence on the impact of drug shop interventions. These reviews were unable to include the full literature on drug shops in Nigeria, limiting our understanding of the nature and quality of PPMV shops. The reviews also indicated that the regulatory and cultural contexts of each country significantly affect drug shop quality and the potential for effective intervention, but delineating country-specific recommendations was beyond the scope of the reviews’ broad conclusions. Thus, a country-level synthesis of available data on PPMVs is needed in order to inform the scale-up of national initiatives, as well as to identify where additional country-specific evidence is needed.

In this article, we comprehensively review the evidence on the characteristics, knowledge and practices of PPMVs in Nigeria in order to assess the strength of the existing evidence base, synthesize what is known about this sector of providers, and identify the remaining gaps in knowledge where evidence is weak or does not exist. The results of the review are used to determine priority areas for research and evaluation that are needed to inform effective PPMV intervention design in Nigeria and highlight opportunities for strengthening PPMV practice within the current regulatory context in the country.

## Methods

We searched the published literature using the following electronic databases: PubMed, Social Science Citation Index, Science Citation Index, and Global Health. Grey literature was identified through web searches (Google Scholar) and searches of relevant international agencies, including the United States Agency for International Development, the UK Department for International Development, World Health Organization, and World Bank. Additional literature was identified through searching the reference lists of included articles. Our search included a combination of terms on “Nigeria” and “drug” or “medicine,” and “retailers,” “dealers,” “shops,” “stores,” “detailers,” “sellers,” “vendors,” “outlets,” “dispensers,” or “dispensaries.”

We included all articles that presented outcome data on PPMVs. We excluded articles on other types of drug retailers, such as pharmacists and itinerant drug hawkers, which are governed by different policy and regulatory statutes than PPMVs. Due to the limited scholarship on this topic, we included all qualitative and quantitative studies regardless of design, and included studies on any outcome within the PPMV sector. We included only articles published in 2000 or after; changes to the scope of practice and monitoring guidelines that affect the role of PPMVs within Nigeria’s health system make studies published after this date of greater relevance to programmatic and policy initiatives on PPMV practice.

Two authors independently screened all titles and abstracts to identify articles for inclusion. Discrepancies in determination of eligibility were decided through consultation with a third author. In order to meet the eligibility criteria, articles had to (1) report at least one PPMV-level outcome, and (2) be published in 2000 or after.

We included all PPMV-level outcomes in the review; outcomes were coded during the data extraction process based on the data reported in the literature. In order to facilitate synthesis of the literature, we grouped related outcomes into categories during the analysis stage. Our results are thus presented five categories: PPMV characteristics, shop quality, knowledge, practice, and compliance. PPMV characteristics included PPMVs’ qualifications and training, the services offered by the shop, drugs stocked and drug prices. Shop quality included the quality of storage facilities and drugs stocked, as well as adherence to general pharmacological guidelines. Knowledge was broadly defined, and included PPMV knowledge of any health area as defined by the study in which the outcome was reported. Given the heterogeneity in how knowledge was measured across studies it was not possible to use a standard definition. Practice included all outcomes related to PPMVs’ dealings with their customers, namely dispensing, client interaction and referral. Finally, we defined compliance as related to the national regulatory requirements and legal scope of practice for PPMVs in Nigeria, including licensing, commodities sold, and services offered. Due to the diversity of outcomes, and variation in definitions of each outcome across the reviewed studies, meta-analysis was not possible. We followed the systematic review guidelines in the Cochrane Handbook for Systematic Reviews of Interventions, including for defining and conducting the search, screening and selecting articles, and extracting data for analysis [32]. No protocol was registered for this review.

Each study was assessed for methodological rigor and bias using the 9-point rigor scale for non-randomized studies developed by the WHO-Johns Hopkins Synthesizing Intervention Effectiveness Project [33]. This scale evaluates studies on several dimensions; inclusion of pre- and post-intervention data, inclusion of a control group or cohort, comparability of the control and treatment groups, random assignment to the intervention and selection for the study, adequate assessment of confounders, and study follow-up.

## Results

### Study characteristics

**Figure 1:**
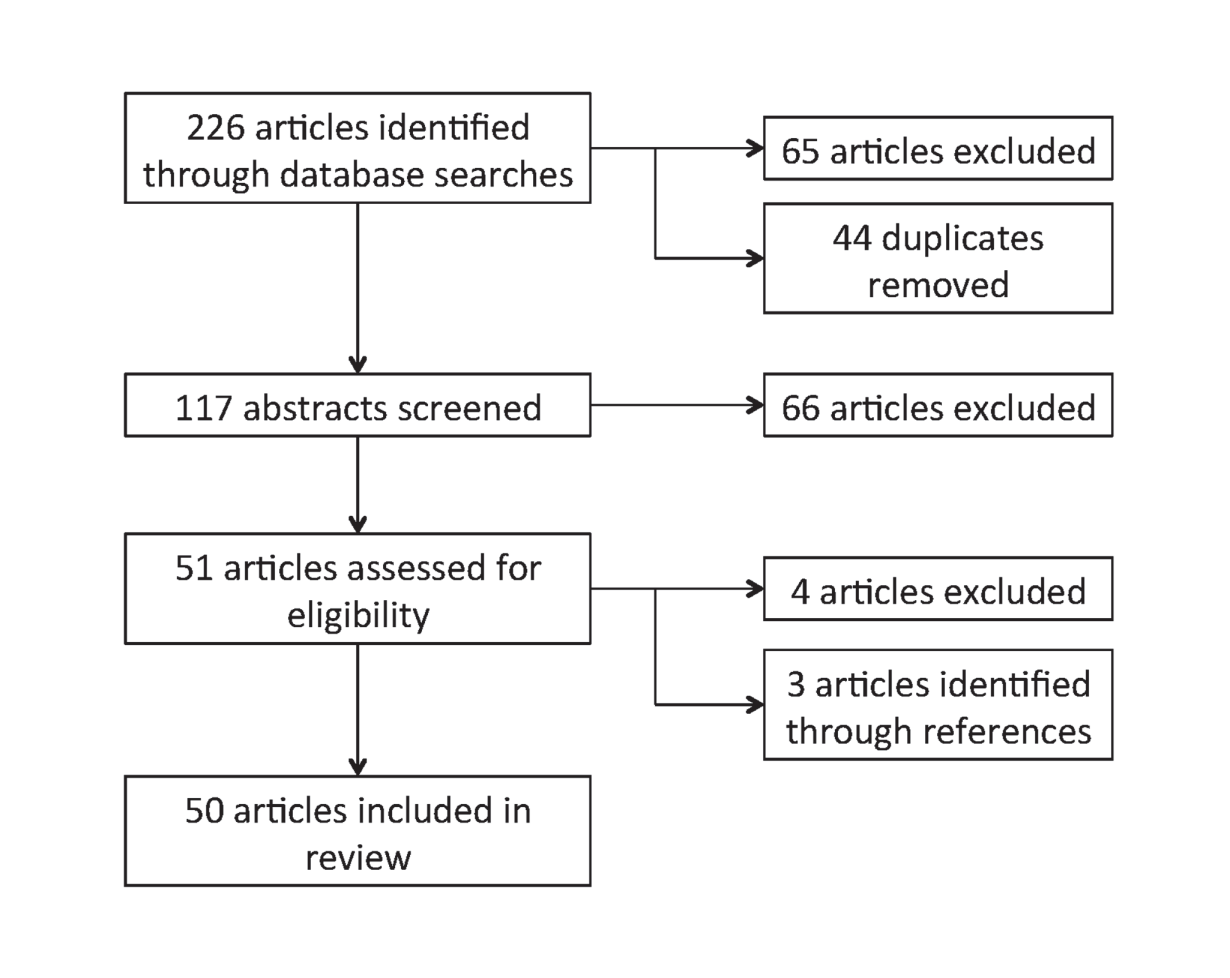
PRISMA study selection flow diagram.

Of the 226 articles identified through the database and website searches, we screened 117 for review and assessed 51 for eligibility. Articles were excluded during these two stages if they did not include PPMV-specific outcomes. Three additional articles were identified through references. A total of 50 articles were included in the review (Fig. 1); of these eight were from the grey literature [34–41]. A full list of the reviewed papers, including health focus area, geopolitical region, data collection method, and categories of outcomes reported, is presented in Table 1.

**Table 1:** Summary of included studies.

Study	Health area	Geopolitical region	Study Design	Rigor score (Max = 9)	PPMV characteristics			Shop quality	Knowledge	PPMV practice			Compliance with legal regulation
					Qualification & training	Services	Drug stock, price			Dispensing	Client interaction	Referral	
ACTwatch 2009	Malaria	National	Cross-sectional; observation (stocking)	1			X	X	X		X		
ACTwatch 2012	Malaria	National	Cross-sectional; observation (stocking)	1	X		X	X	X		X		
Adedeji et al. 2011	Malaria	South West	Cross-sectional	1						X			
Aguwa et al. 2010	Child illness	South East	Cross-sectional	0	X		X		X	X		X	
Ajayi et al. 2002	Malaria	South West	Cross-sectional	1					X	X	X		
Akiode et al. 2010	Pharmaco-vigilance	South West; North Central	Cross-sectional	1			X						
Akuse et al. 2010	Malaria	North West	Cross-sectional	0	X					X		X	
Aniebue et al. 2010	Other	South East	Cross-sectional	0				X	X				
Auta et al. 2012	Other	North Central	Cross-sectional	0					X				
Awodele et al. 2012	Pharmaco-vigilance	South West	Pre/post	4	X				X				
Awofisayo et al. 2008	Pharmaco-vigilance	South South	Cross-sectional	0	X								X
Berendes et al. 2012	Malaria	North West	Cross-sectional	1	X		X		X	X			
Brieger et al. 2002	Child illness	South West; South East	Pre/post	3						X			
Brieger et al. 2004	Other	South West	Observation (interaction)	1							X	X	
Brieger, 2007	Pharmaco-vigilance	South West	Qualitative	0			X						
Chukwuocha et al. 2013	Malaria	South East	Cross-sectional	1	X		X		X		X		
Ebong et al. 2012	Malaria	South South	Cross-sectional	1					X	X			
Enwere et al. 2014	Malaria	South West	Cross-sectional	1					X	X			
Erhun and Osagie 2004	Malaria	South West	Cross-sectional	1			X				X		
Fajola et al. 2011	Child illness	South West	Cross-sectional; observation (interaction)	1	X						X		X
Fayemi et al. 2010	FP/STIs	South West	Cross-sectional	0	X		X		X			X	
FHI360 2013	FP/STIs	South West	Cross-sectional	0	X		X		X				X
Greer et al. 2004	Malaria	South East	Pre/post	7					X	X			
Herbert et al. 2013	FP/STIs	South West; North Central	Qualitative	0							X		
Idowu et al. 2006	Malaria	South West	Cross-sectional; laboratory analysis	0	X		X	X		X			
Jimmy et al. 2000	Malaria	South South	Cross-sectional	0	X		X				X		
Livinus et al. 2009	Malaria	North West	Pre/post; mystery client	4	X		X		X	X	X		
Mangham et al. 2011	Malaria	South East	Cross-sectional	2	X		X		X		X		
Nduka et al. 2013	Malaria	South East	Cross-sectional	1	X		X		X				
Obi et al. 2010	TB	South East	Cross-sectional	1	X				X			X	
Obitte et al. 2007	Other	South East	Cross-sectional	0	X			X	X				X
Okeke and Uzochukwu 2009	Malaria	South East	Pre/post; client exit interview	3	X				X	X	X		
Okeke et al. 2006	Malaria	South East	Qualitative	0	X				X	X	X	X	
Okonkwo and Okonkwo 2010	FP/STIs	North Central	Cross-sectional	0		X	X				X		X
Oladepo et al. 2007	Malaria	South West; South East; North Central	Cross-sectional; observation (stocking); qualitative	1	X		X		X				
Oladepo et al. 2008	Malaria	South West, South East, North West	Cross-sectional	0			X		X				
Oladepo et al. 2011	Malaria	South West; South East; North Central	Cross-sectional; observation (stocking)	1	X		X	X	X		X	X	
Onwujekwe 2010b	Malaria	South South	Cross-sectional; client exit interview	0			X						
Onwujekwe et al 2009	Malaria	South East	Laboratory analysis	0				X					
Onwujekwe et al. 2010	Malaria	South East	Cross-sectional	1	X		X			X	X		
Onwujekwe et al. 2011	Malaria	South East	Cross-sectional	2						X			
Onyeneho and Chukwu 2010	TB	South West, South East, South South	Cross-sectional; qualitative	1	X				X			X	
Oyeyemi et al. 2014	Malaria	South West	Cross-sectional	1	X		X	X					X
Palafox et al. 2012	Malaria	National	Cross-sectional	1			X						
Spaid et al. 2011	FP/STIs	National	Qualitative	0							X	X	X
Tekobo et al. 2004	Malaria	South West	Cross-sectional	1	X				X	X			X
Tobin-West and Adeniji 2012	Malaria	South South	Cross-sectional	1	X			X	X	X		X	
Ujuju et al. 2014	FP/STIs	South West; South East; North West; North Central	Mystery client; qualitative	2							X	X	X
Um et al. 2007	Malaria	North West	Cross-sectional	1	X				X	X			
Uzochukwu et al. 2014	Child illness	South East	Cross-sectional	1							X		X

**Figure 2:**
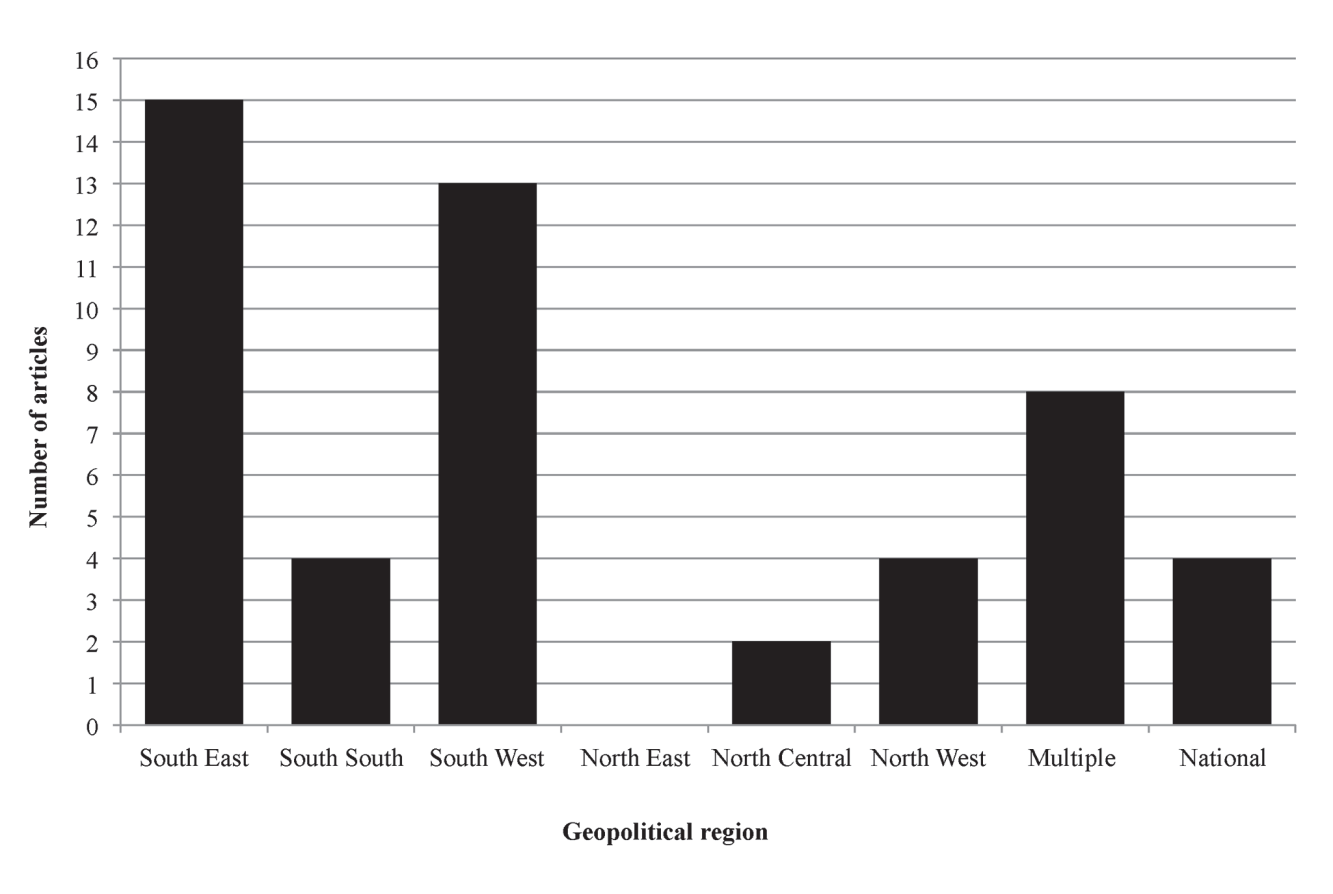
Geographic location of included studies.

**Figure 3:**
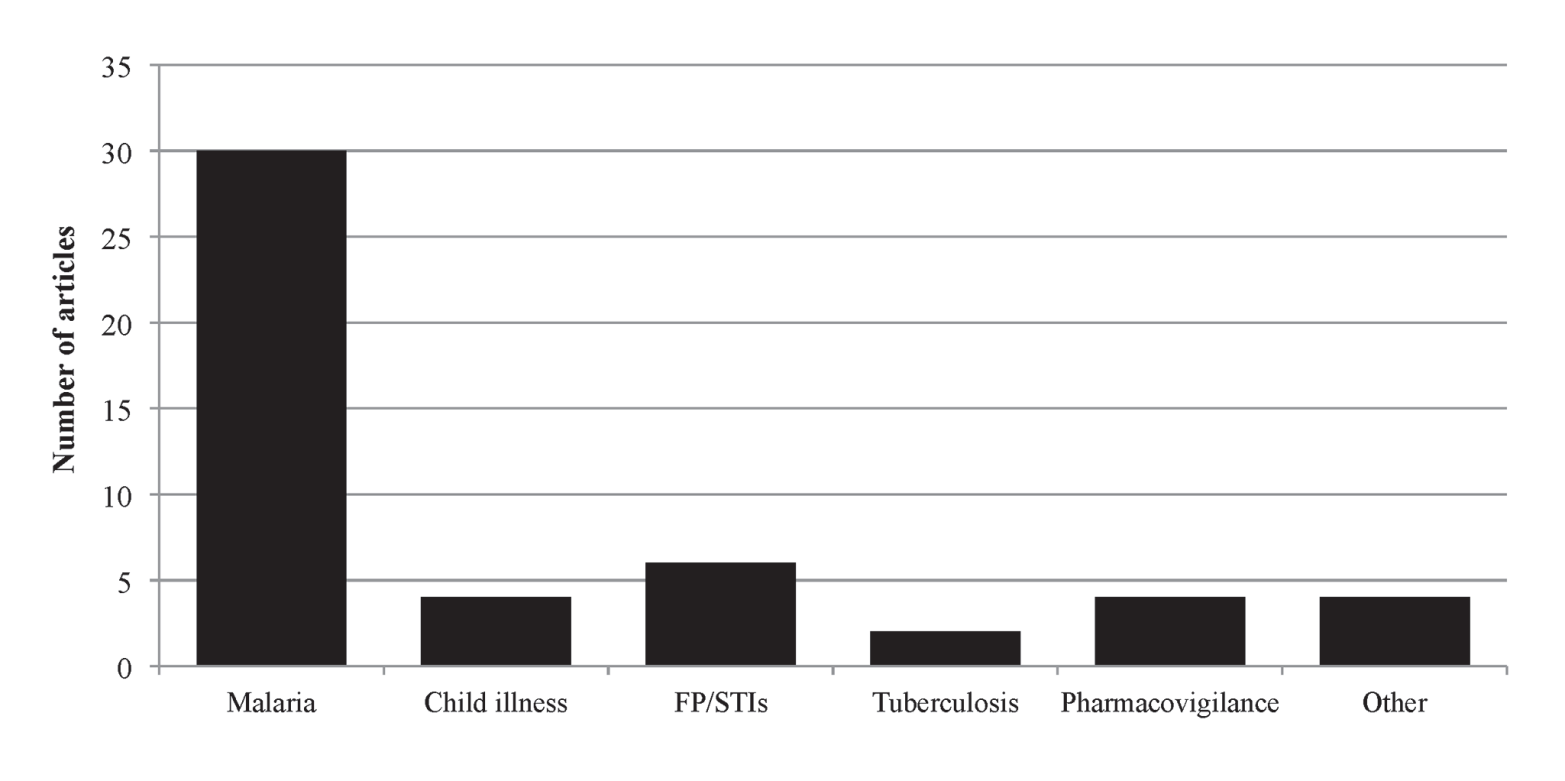
Health area of focus of included studies.

The existing literature on PPMVs is focused largely in Nigeria’s southern geopolitical regions, with few studies conducted in northern regions (Fig. 2, Table 1). Eight studies covered multiple regions: two of these covered multiple regions in the South, and the remaining six covered at least one region in the South and one in either North West or North Central Nigeria (Table 1). Nationally representative results are sparse; all nationally representative data are from the ACTwatch surveys, which track the availability and pricing of anti-malarial drugs [37–39]. The majority of studies focused exclusively on malaria-related outcomes (Table 1). Other health conditions were largely absent from the PPMV literature, although there are a small number of studies focusing on family planning or sexually transmitted infections (FP/STIs) (Fig. 3, Table 1). Only four studies addressed child health (Table 1), whereas the majority focused on adult health.

The majority of studies relied on cross-sectional surveys (n = 38, Table 1). Only six studies evaluated the impact of interventions, five of which used a pre-post design [34,42–45] and one of which used post-intervention cross-sectional data [40]. Other methods employed included qualitative interviews (n = 7), observations of PPMV-client interactions (n = 2) or PPMV stocking practices (n = 4), mystery clients (n = 2), client exit interviews (n = 2), and laboratory analysis (n = 2). Ten studies presented outcomes obtained through two different data collection methods, and one study presented outcomes obtained through three data collection methods (Table 1).

### PPMV Characteristics

Studies evaluating the characteristics of PPMVs and their shops included various outcomes on the educational background and training of PPMVs, and the services and medicines offered at their shops. These studies highlight the wide range of health areas across which PPMVs provide services.

**Qualifications and training.** Twenty-seven studies reported on PPMVs’ level of education and/or source of training (Table 1). These studies indicate that many PPMVs have higher than the customary level of primary education. The percentage of PPMVs with tertiary education ranged from 14% in Anambra state [46] to 57% in Jigawa [47]. The majority of studies found that between 7% and 20% of PPMVs had only primary education [42,43,47–56], though rural PPMVs were less educated than their urban counterparts [44,50]. It is worth noting that many PPMVs have apprentices or other employees who staff the shop when the owner is away [21]; no studies compared the educational levels of these staff with the owner.

A substantial percentage of PPMVs have received formal medical training, ranging from 14% in Enugu [57] to 20% in Rivers and Kano [54,58] and 32% in Lagos [59]. Prior or concurrent employment in a health center was also common; 29% of surveyed PPMVs in Oyo had worked at a health facility [41], with rural PPMVs twice as likely than those in urban areas to be dually-employed [60]. However, apprenticeships with another PPMV remains the primary source of training for the majority of PPMVs [35,41,61,62]. PPMVs’ were found to have an average of between 5.4 and 15.5 years of experience [41,42,46,55,59,61].

**Services.** The reviewed studies show that PPMVs provide medicines and services for a wide variety of health needs, including malaria, respiratory infections, diarrhea, common cough and cold, tuberculosis, and reproductive health (Fig. 3). However, holistic information about the full range of illnesses treated or products sold at PPMV shops is not available because all of the included studies were designed to evaluate outcomes for specific diseases or health needs. One study asked PPMVs about their primary role, and reported that PPMVs mainly sell medicines, though some also provide additional services including treating minor ailments and referring complicated cases [63].

**Drug stock and price.** Twenty-two studies assessed drug stocks for specific illnesses or health needs at PPMV shops (Table 1); the lack of studies assessing the full stock at PPMV shops prevents identification of the range of illness for which PPMVs stock products. Three-quarters of these studies focused on stocking practices for anti-malarial medicines (Table 1). Studies conducted after the 2005 change in treatment guidelines for malaria found that stocking of ACTs has increased over time, from 37% [37] in 2009 to 54% in 2011 at a national level, following the implementation of the Affordable Medicine Facility-malaria (AMFm) pilot, which aimed to increase the availability of ACTs [38]. Smaller scale studies found around 60% [49,59] of PPMVs stocking ACTs, reaching up to 97% in some states [57]. However, many studies found that PPMVs continued to stock non-recommended drugs for malaria including chloroquine and artemisinin monotherapies [35–38,43,47,62,64].

PPMVs also stock a number of sexual and reproductive health products. The data in this area, which are based on small scale studies, showed that male condoms and treatments for sexually transmitted infections (STIs) are more commonly stocked than emergency contraceptives, female condoms and birth control pills [61,63]. Another study found that 13% of PPMVs sold injectable contraceptives even though they are prohibited from giving injections [41]. In contrast, while PPMVs are allowed to sell misoprostol to treat postpartum hemorrhage, the single study on this topic found that no shops stocked misoprostol six months after the drug was approved for sale [65]. PPMVs reported that stocking decisions were motivated by regulations, religion, side effects, effectiveness, brand reputation, ability to procure supplies, and consumer demand [37,48,58].

All of the evidence on drug prices at PPMVs comes from the malaria literature. These studies showed that prices for anti-malarials at PPMVs are lower than at all other sources except for public sector facilities [37–39,57,66]. The most recent national data on anti-malarial pricing at PPMVs indicated that percentage mark-ups were similar across different anti-malarials, ranging from 22%–40% (around USD 0.19 to 0.72), lower mark-ups than those charged by either pharmacies or private health facilities [39]. Due to the lack of studies on prices or profit margins for other types of drugs sold by PPMVs, it is not possible to assess relative profits or the importance of different health areas for PPMVs’ overall business model.

### PPMV Shop Quality

Nine studies addressed the quality of PPMV shops (Table 1). The evidence on quality focuses primarily on drug quality, particularly the quality of anti-malarial drugs stocked. Drug inventory assessments have shown that the majority of anti-malaria drugs stocked by PPMVs had a NAFDAC number verifying the manufacture information, and that few were expired [38,56,59]. Nationally, only 2% of PPMVs stocked expired anti-malarial medications [37], but regional studies showed more PPMVs carried expired drugs, ranging from 3% of PPMVs in Kaduna and Oyo to 15% of PPMVs in Enugu [62]. Studies on drug quality also indicated that PPMVs carry relatively low quality anti-malarials; chemical analysis studies found that roughly half of anti-malarial drugs stocked in PPMV shops were sub-standard [53] [67]. This was a significantly higher percentage than stocked in pharmacy shops and public primary healthcare centers in the same area [67], though PPMVs with higher levels of education were more likely to sell quality drugs [53].

Assessments of the equipment and drug storage facilities at PPMVs [37,38,68], found that most shops could properly store malaria medicines [37,38] but not medications that require stricter temperature controls [68]. One study assessed PPMV shop quality as measured by the utilization of universal precautions for infection prevention, and found that 43% of PPMVs improperly disposed of sharps after administering injections (which is outside their legal scope of practice), and only 1% of PPMVs used gloves, properly disposed of sharps, and referred customers to avoid contact with blood [69].

### PPMV knowledge

Knowledge was the most widely reported outcome in this literature; 28 articles reported on PPMV knowledge of malaria, child health, family planning, tuberculosis (TB) or pharmacological standards (Table 1). PPMV knowledge was limited, although PPMV knowledge varied across health topics and geographic regions. In general, PPMVs appeared to have better knowledge of the causes and symptoms of illnesses, but poorer awareness and knowledge of correct treatment guidelines.

**Malaria knowledge.** The majority of studies (n = 19) addressing PPMV knowledge focused exclusively on malaria (Table 1). Although knowledge indicators were not comparable across studies, results indicated that PPMVs have good recognition of malaria symptoms [46,55,70]. The majority of PPMVs in Enugu and Lagos states correctly identified the cause of malaria [55,70]; however, only 8% of surveyed PPMVs responded correctly in Anambra state [46], and PPMVs frequently identified incorrect causes of malaria [55,70]. PPMVs have poorer knowledge of proper malaria treatment; nationwide, 65% to 69% of PPMVs could name at least one malaria danger sign requiring referral to a health facility [37,38]. Smaller studies found that the percentage of PPMVs who properly identified danger signs of severe malaria ranged from 14% in Jigawa [47] to 71% in Anambra [46].

For knowledge of recommended malaria treatment, we focused on studies conducted after the 2005 revision of the national malaria treatment guidelines. State-level studies from this period found that between 16% and 80% of PPMVs were aware that the guidelines had been revised [35,62] and that between 6% and 51% were aware of the content of the revisions (i.e. ACTs replacing chloroquine as first-line treatment for uncomplicated malaria) [35,47,49,56,57,62,71]. The national ACTwatch surveys found that in 2011, 51% of PPMVs knew the correct first-line treatment for malaria, an increase from 14% in 2009 [37,38]. PPMV knowledge of the correct ACT dosages for adults and children also increased from less than ten percent in 2009 [37] to 53% (child dosage) and 79% (adult dosage) in 2011 [38]. PPMVs were found to have poorer knowledge of malaria treatment than public health facility staff [37,38,57] and pharmacists [37,38,71,72], but comparisons between PPMVs and Community Health Extension Workers (CHEWs) showed varying results as to which group had better knowledge [37,38,71].

**Other health knowledge.** Nine studies assessed PPMV knowledge of non-malaria health topics (Table 1); all found low knowledge of illness identification and proper drug dispensing. The only article addressing PPMV knowledge of child health for illnesses other then malaria focused on diarrhea, finding that PPMVs were aware of the causes and signs of diarrhea, but were unaware of diarrhea prevention and use of oral rehydration salts for treatment [48].

PPMVs also had poor knowledge of the causes of TB, the correct definition of chronic cough, and the correct duration of TB treatment [50,51]. Comparisons of TB knowledge between urban and rural PPMVs produced inconsistent results across the two studies, with one showing that urban PPMVs had better knowledge [50] and the other that rural PPMVs had better knowledge [51].

Two studies, both conducted in the South West region, found high levels of awareness of family planning methods: 89% of PPMVs knew about injectable contraceptives [41] and 72% reported that they knew emergency contraceptives could be used within three days of sexual intercourse [61]. However no studies assessed PPMV knowledge of the proper use, or the benefits and risks, of different family planning methods.

Studies also found that PPMVs had generally low knowledge of pharmacological and safety guidelines [42,68,69,73]. PPMVs had poor knowledge of drug administration, including understanding of drug interactions, contraindications, and side effects [73], and limited knowledge and awareness of universal precautions for infection prevention [69].

### PPMV practice

Studies assessing PPMV practice addressed dispensing practices, client interaction, referral to health facilities, and compliance with legal requirements. As with knowledge, the findings were variable, but generally indicated poor practices.

**Dispensing.** The 18 articles that assessed PPMV drug dispensing practices (Table 1) found that PPMVs generally do not follow treatment guidelines. Observations of PPMV dispensing practices indicated that the majority of anti-malarial drug sales were of non-recommended drugs [74]; and a study using clinical vignettes found that only half of PPMVs recommended the proper treatment for malaria [16]. Studies based on PPMV self-reports indicated similarly poor dispensing practices. Three studies conducted prior to the 2005 malaria treatment guideline revisions found that fewer than 20% of PPMVs reported selling the correct dosage of chloroquine [45,53,55]. Following the 2005 revisions, the percentage of PPMVs who reported dispensing ACTs for uncomplicated malaria ranged from near zero in Anambra and Enugu states [64,70] to 69% in urban Rivers state [71]. Between 46% and 77% of PPMVs reported correct dispensing of sulfadoxine pyrimethamine for pregnant women [56,71]. A single study of dispensing practices for non-malaria illnesses found that 69% of PPMVs reported ORS as their first-line treatment for diarrhea in under-fives [48]. The reliance of these studies on self-reported data may overstate rates of proper treatment; a study comparing self-reported to observed dispensing practices for child malaria found that PPMVs under-reported dispensing of improper treatments [75].

**Client interactions.** Nineteen articles assessed the interactions between PPMVs and their clients (Table 1), focusing on drug choice, consultation about the illness, and instructions regarding drug administration. Interactions about drug choice, and the majority of drug sales, were largely driven by customer demand for specific drugs [37,38,44,49,70,75,76]. Although some PPMVs report that customers ask for recommendations on drug choice, particularly for sick children [75] and anti-malarial drugs [37,38], observational data showed that PPMVs only suggested drug choices in 31% of encounters [21], and that between 58% and 69% of PPMVs sold drugs as requested without asking the customer for any clarification [21,60]. Very few customers requested drugs based on prescriptions [49,58,70,75]; observational data found that between zero and 9% of clients brought a prescription to the PPMV shop [21,60], with higher percentages in rural areas [60].

Customer willingness to pay also impacted drug choice interactions. Qualitatively, PPMVs reported that their decisions about drug type and dosage were influenced by their perceptions of a customers’ ability or wiliness to pay [70], a practice that was corroborated by a mystery client survey [43]. Data from customer exit surveys showed that customers seeking care for children purchased an average of 6.8 drugs, with 55% purchasing non-essential drugs to treat malaria, acute respiratory infections, and diarrhea, resulting in an average additional and unnecessary expense of USD 1.09 to 2.19 per illness episode [77].

Customers infrequently consulted PPMVs about illness diagnosis, and PPMVs reported that they infrequently asked their customers about their illness history or conducted examinations [62,64,70,75]. Observational data and client exit surveys indicated that discussions of illness history occurred in 19% to 32% of encounters [21,57], although nearly a quarter of PPMVs reported that they would ask to see the sick child if she was not present at the shop [75]. It was also uncommon for PPMVs to instruct customers on the proper administration or side effects of purchased drugs, or to discuss referral for further treatment [21,43,57,75,76]. Studies on family planning services at PPMVs found similarly limited counseling for customers. Mystery client visits indicated that less than 15% of customers purchasing birth control pills were informed about side effects [40,78]. Some PPMVs expressed the need to maintain good communications, discretion, and confidentiality when dispensing family planning products to young customers [63], however others indicated that they may exclude youth and unmarried customers from receiving family planning services [79].

**Referral.** Eleven studies reported on referral practices among PPMVs, many of which found low rates of referral. The majority of these studies relied on PPMV self-reports; a single study used direct observation of PPMV-client interaction to document referral practices across all health conditions, and found that only 0.4% of customers were referred [21].

Two studies on malaria found wide variation in referral practices; the percentage of PPMVs who reported that they would refer a customer in the case of serious illness, or if there was no improvement after initial treatment, ranged from 5% [62] to 68% [56]. For family planning, 51% of PPMVs self-reported referring clients to health facilities for related services [61]. Yet two mystery client studies found that only 30% to 45% of PPMVs referred first-time users, and about 50% referred current users experiencing contraceptive-related complications [40,78]. Low rates of referral were also documented for customers with TB. In Enugu, only 5% of urban and 18% of rural PPMVs reported referring customers with “long-lasting” cough, and none of the referrals were to registered DOTS centers [50]. A second study found a higher percentage of PPMVs who reported ever having referred a customer for a prolonged cough (77%), however the average wait time prior to this referral was 39 days [51].

Interviews with PPMVs show that reasons for referral included the inability to treat an illness, lack of improvement following initial treatment, severe illness, and the need for diagnostic tests [52]. Reasons for not referring included the inaccessibility or understaffing of health centers [70] and the fear of potentially losing customers [78].

### Compliance with legal regulations

Ten studies on PPMV compliance with legal regulations focused on licensing, drug stocking, and drug sale requirements (Table 1). Evidence on PPMV regulatory compliance is more robust in the area of family planning than in other health areas.

**Licensing.** Several studies indicated that many shops operate without the proper licenses. In Lagos, only 6% of shops had a “practicing” license [59]and 38% were registered with PCN [55], while in Akwa Ibom 20% of shops were licensed [80].

**Commodities.** Between 60% and 70% of shops stocked medications they are legally prohibited from selling [60]. One study on the services PPMVs provided for sick children showed that 60% received an antibiotic [77] despite the fact that the sale of antibiotics is outside PPMVs’ current scope of practice. This may be due to a lack of knowledge regarding legal scope of practice; one study found that awareness among PPMVs of the rules restricting antibiotic sales varied from 79% in rural areas to 6% in urban areas of Oyo [60]. PPMVs have also been found to sell drugs re-packaged out of their original container, in contrast to the legal guidelines restricting them to the sale of pre-packaged drugs [68].

**Services.** Studies show that PPMVs also provide diagnostic and treatment services that fall outside their legal scope of practice. Two studies on malaria found that PPMVs largely comply with prohibitions on conducting diagnostic tests, but do not comply with the regulation against giving injections [55,77]. Close to half of PPMVs in Lagos reported administering injections for malaria treatment [55], and a study of child health showed that 73% of children attending PPMV shops received an injection [77].

Studies on family planning also found that many PPMVs do not comply with current regulatory guidelines; between 9% and 17% of PPMVs sold oral contraceptives to mystery clients posing as first time pill users, and there were low rates of referral for family planning-related complications [40,78]. PPMVs also provided a number of family planning and reproductive health services beyond their legal scope of practice. In South West Nigeria, 15% of PPMVs reported providing family planning injections [41]. PPMV clients interviewed in the North Central region reported receiving abortion care, post-abortion care, and treatment of sexually transmitted infections at PPMV shops [63].

### PPMV Interventions

Only six studies evaluated the impact of interventions targeting PPMVs [34,40,42–44,81]. The majority of interventions focused on training activities. All interventions resulted in improved knowledge, while the evidence was more mixed regarding the impact of interventions on improving PPMV practice. In Kebbi, a training intervention increased PPMV knowledge of first-line anti-malarial treatment and referral for symptoms, but did not improve proper dispensing for malaria treatment [43]. A training program on pharmacovigilance in Ekiti state increased PPMV knowledge of the concept of adverse drug reaction reporting, but did not examine the impact on PPMV practices [42]. More comprehensive interventions complementing training with demand- and supply-side components (e.g. job aids, completion certificates, community mobilization, assured supply) increased both PPMV knowledge of and proper dispensing practices for malaria [34,44]. An intervention in Oyo, Abia, and Enugu states increased proper prescribing practices for child malaria [81], and another intervention improved PPMV-client interactions, increasing the percentage of PPMVs who took customers’ illness history [44].

The two ACTwatch outlet surveys include baseline [37] and endline [38] reports for the evaluation of the AMFm pilot intervention in Nigeria which provided a supply-side subsidy designed to increase the affordability and availability of ACTs [38]. As noted above, the percentage of PPMVs stocking ACTs increased from 37% to 54% over the course of the pilot. The median sale price of quality-assured ACTs at PPMVs also decreased, and the market share of ACTs among PPMVs’ anti-malarial sales increased from 6.3% to 19.0%. PPMVs’ knowledge of ACTs as the recommended first-line treatment for malaria and the correct dosages for adults and children also improved [37,38].

### Methodological rigor and bias

Although we found 50 published articles that included PPMV-specific outcomes on a wide range of topics, our ability to draw strong conclusions from this body of literature is limited due to the methodological weakness of the evidence base and the diversity of definition and measurement across outcomes. The included studies on PPMVs primarily employed cross-sectional data collection with convenience sampling strategies, and therefore the majority of studies scored below a 2 on the 9-point WHO-Johns Hopkins scale, indicating high levels of potential bias. Only the five articles that included pre- and post-intervention data scored a 3 or higher on the scale, and 18 studies did not meet any of the criteria included in the scale (Table 1). Weaknesses in study design were compounded by differences across studies in survey questions and indices used to measure similar outcomes, such as knowledge. Furthermore, results on a number of outcomes, such as stocking, dispensing and referral practices, largely rely on PPMV self-reported data that are prone to a number of biases.

## Discussion

PPMVs are an important source of health care in Nigeria, providing drugs and services across a wide range of health areas—from malaria and diarrhea to respiratory infections, tuberculosis, and sexual and reproductive health. Given the scale and scope of this sector, PPMV shops could be part of an effective strategy to increase access to essential health commodities. Acknowledging this, health policies and intervention pilots in Nigeria are increasingly incorporating PPMVs as key community-level health providers. Our review highlights several areas where services provided at PPMV shops need to be improved in order to capitalize on the potential value of this sector and a number of research questions that will need to be answered in order to achieve this.

The available evidence shows that PPMVs provide low quality health services. PPMVs stock poor quality medicines and stock a large number of medicines they are prohibited from selling. In addition, the evidence from the malaria literature indicates that their limited knowledge of proper treatment practices results in frequent sales of inappropriate and ineffective medicines and the sale of drugs in incorrect doses. Although only one study directly measured the cost of irrational dispensing, the fact that PPMVs dispense inappropriate medications suggests that they may be causing unnecessary expenditures for customers, in addition to improperly treating illnesses. Customer demand for improper treatment further contributes to poor dispensing practices; PPMVs are infrequently consulted about illness diagnosis or drug choice.

Studies of malaria and family planning services also show that PPMVs frequently provide services beyond their legal scope of practice. The prevalence of PPMVs offering medicines and healthcare services they are prohibited from providing raises concerns about the quality of these drugs and services. This is particularly true as PPMVs are not typically trained on counseling and often do not provide customers with information about drug administration or side effects, or referral for further treatment. Restrictions on the drugs PPMVs are allowed to sell, without adequate enforcement capacity, may increase stocking of fake and substandard drugs by limiting the options PPMVs have for purchasing high quality medicines. In addition, PPMVs are unable to receive training on how to properly dispense these medicines, or how to safely provide services such as injectable family planning, given the current legal restrictions.

Although current research documents poor quality at PPMV shops, the studies included in this review also suggest that there are opportunities to improve the quality of medicines and services provided by PPMVs. First, although the law requires that PPMVs have only primary schooling, the majority of studies found that PPMVs have significantly higher levels of education and that a substantial percentage are trained health professionals, suggesting that they could be more effectively targeted for quality improvement. Second, the available evidence suggests that well-designed interventions can improve the quality of treatment PPMVs provide for common illnesses. Integrated interventions that address both supply and demand-side factors, and that do not rely solely on training, are more effective in changing PPMV practice than training-only interventions. This is in line with a broader review of interventions for informal providers that found that the most effective interventions were those that targeted market or institutional environments, in addition to providing training [82].

There are also successful models elsewhere in Sub-Saharan Africa for segmenting the drug retail sector and implementing large-scale interventions to improve the quality of services provided by retail outlets and to better integrate them into the formal health system. In Kenya, government-run training programs for drug shop owners increased proper dispensing of malaria treatments [5,6,83]. In Tanzania, a national drug shop accreditation program increased the quality of care in drug stores, closed down unqualified drug shops, and resulted in greater accessibility of recommended malaria treatments at the community level [7,84].

However, further research is needed in several areas to inform effective, scalable interventions to improve the services provided by PPMV shops in Nigeria. First, too little is known about the nature and quality of the full range of services and products offered at PPMV shops. The vast majority of evidence focuses exclusively on malaria services for adults, despite the indication that PPMVs are serving a much wider range of illnesses and clients. This is a critical gap in our understanding both of the health services that PPMVs provide, and of the business and financial incentives under which PPMVs operate. Prior reviews of drug shop interventions indicate that profit motivations impact shopkeeper behavior and intervention effectiveness [30]. Thus, additional research is needed to understand the range of products and services provided by PPMVs, including their stocking behavior, sales volumes and profit margins across disease areas. At the same time, there is a need to assess the quality of the range of services provided by PPMVs, as well as their knowledge of and capacity to provide safer and higher quality care in the relevant health areas. Of particular importance is research that assesses PPMV knowledge and practice for child illnesses.

Research should also focus on testing and evaluating mechanisms for improving the quality of care at PPMV shops within the policy and regulatory context of Nigeria, and the particular role that PPMVs play in the broader health system. Although early evidence suggests that training can improve knowledge, it is clear that this does not always translate into improved dispensing practices. For example, even with the national-level AMFm mechanism, which has improved availability, affordability, and knowledge of ACTs, the market share of non-recommended anti-malarials remains high. This is indicative of the need for further evidence on how PPMVs’ incentives for dispensing drugs are currently structured and how they might be changed to improve quality of care, particularly in a context where customer demand plays an important role in retail transactions. However, the AMFm pilots also indicate that supply-chain interventions, such as subsidies, can potentially improve stocking of recommended treatments. There is also little or no evidence on the sustainability of intervention effects over time, the efficacy of monitoring and enforcement mechanisms for quality assurance in the PPMV sector, or interventions to improve the quality of non-malaria health services. In addition, there is very little evidence on how PPMVs’ services compare to those of other healthcare providers, making it difficult to assess and understand the role of PPMVs within the broader health system. Further research on the role of PPMVs can improve integrated interventions.

### Limitations

There are several limitations to this review. Given the absence of more rigorous studies, we chose to include both non-experimental and qualitative studies, therefore limiting our ability to draw strong conclusions. The studies focus on a narrow set of service provision outcomes and health areas, and focus almost exclusively on services for adults, further limiting our ability to draw conclusions about the overall quality of care provided at PPMV shops, notably for children. In addition, the majority of the studies are located in the country’s Southern region. Health-seeking behavior, and the role of PPMVs in local health systems, may vary significantly across Nigeria’s diverse regions[9,12,19,20], therefore limiting the generalizability of our findings.

### Conclusions

PPMVs provide a source of access to essential medicines throughout Nigeria, across a wide range of disease areas. Although the quality of services provided by PPMVs is poor, there is some evidence that PPMV knowledge and practice can be improved through interventions, and particularly interventions that go beyond training such as to include demand-side interventions or supply chain support and subsidies. The weakness of the evidence base on PPMVs is, however, a limiting factor in the development of innovative and comprehensive interventions to improve PPMV practice. Given the size of the PPMV sector and its importance in providing services to rural and low-income populations, this is a missed opportunity to improve the coverage and quality of the health system. Future research in this area should adopt a more comprehensive, multi-disease area approach to understanding PPMV practice, and focus on the evaluation of interventions to improve the quality of PPMV services across a range of outcomes.

## Supporting Information

S1 PRISMA Checklist (DOC)
